# Proof of Concept for Genome Profiling of the Neurofibroma/Sarcoma Sequence in Neurofibromatosis Type 1

**DOI:** 10.3390/ijms251910822

**Published:** 2024-10-09

**Authors:** Ilenia Rita Cannizzaro, Mirko Treccani, Antonietta Taiani, Enrico Ambrosini, Sabrina Busciglio, Sofia Cesarini, Anita Luberto, Erika De Sensi, Barbara Moschella, Pierpacifico Gismondi, Cinzia Azzoni, Lorena Bottarelli, Giovanna Giordano, Domenico Corradi, Enrico Maria Silini, Valentina Zanatta, Federica Cennamo, Patrizia Bertolini, Patrizia Caggiati, Davide Martorana, Vera Uliana, Antonio Percesepe, Valeria Barili

**Affiliations:** 1 Medical Genetics, Department of Medicine and Surgery, University of Parma, 43126 Parma, Italy; 2 Human Nutrition Unit, Department of Food and Drug, University of Parma, 43125 Parma, Italy; 3 Medical Genetics, University Hospital of Parma, 43126 Parma, Italy; 4 Pediatric Clinic, Pietro Barilla Children’s Hospital, Department of Medicine and Surgery, University of Parma, 43126 Parma, Italy; 5 Pathology Unit, Department of Medicine and Surgery, University of Parma, 43126 Parma, Italy; 6 Cytogenetics, Molecular Genetics and Medical Genetics Unit, Toma Advanced Biomedical Assays, 21052 Busto Arsizio, Italy; 7 Pediatric Hematology Oncology Unit, University Hospital of Parma, 43126 Parma, Italy

**Keywords:** neurofibromatosis type 1, malignant peripheral nerve sheath tumor, MPNST, tumor progression, genomic signature, whole exome sequencing (WES)

## Abstract

Neurofibromatosis type 1 (NF1) is an autosomal dominant genetic disorder characterized by the predisposition to develop tumors such as malignant peripheral nerve sheath tumors (MPNSTs) which represents the primary cause of death for NF1-affected patients. Regardless of the high incidence and mortality, the molecular mechanisms underneath MPNST growth and metastatic progression remain poorly understood. In this proof-of-concept study, we performed somatic whole-exome sequencing (WES) to profile the genomic alterations in four samples from a patient with NF1-associated MPNST, consisting of a benign plexiform neurofibroma, a primary MPNST, and metastases from lung and skin tissues. By comparing genomic patterns, we identified a high level of variability across samples with distinctive genetic changes which allow for the definition of profiles of the early phase with respect to the late metastatic stages. Pathogenic and likely pathogenic variants were abundant in the primary tumor, whereas the metastatic samples exhibited a high level of copy-number variations (CNVs), highlighting a possible genomic instability in the late phases. The most known MPNST-related genes, such as *TP53* and *SUZ12*, were identified in CNVs observed within the primary tumor. Pathway analysis of altered early genes in MPNST pointed to a potential role in cell motility, division and metabolism. Moreover, we employed survival analysis with the *TCGA* sarcoma genomic dataset on 262 affected patients, in order to corroborate the predictive significance of the identified early and metastatic MPNST driver genes. Specifically, the expression changes related to the mutated genes, such as in *RBMX*, *PNPLA6* and *AGAP2*, were associated with reduced patient survival, distinguishing them as potential prognostic biomarkers. This study underlines the relevance of integrating genomic results with clinical information for early diagnosis and prognostic understanding of tumor aggressiveness.

## 1. Introduction

Neurofibromatosis type 1 (NF1) is a neurocutaneous autosomal dominant disorder, with a birth incidence of 1:3500, caused by a heterozygous inactivating mutation in the tumor suppressor NF1 gene (17q11.2) [1,2,3]. Clinical manifestations of NF1 are highly variable, mainly consisting in multiple café-au-lait macules (CALMs), axillary and inguinal freckling, Lisch nodules, and cutaneous and plexiform neurofibromas. Some individuals also develop skeletal abnormalities, intellective disabilities and attention deficits, brain tumors and malignant peripheral nerve sheath tumor (MPNST) [2,4].

Indeed, *NF1* is a tumor suppressor gene which encodes for Neurofibromin, a GTPase-activating protein (GAP) for the RAS family of proto-oncogenes, which induces a conformational change that stimulates the intrinsic GTPasic activity of the Ras protein [1,5]. Loss of neurofibromin leads to persistent RAS signaling and uncontrolled cellular growth through downstream RAF, MEK, and ERK signaling [6].

Malignant peripheral nerve sheath tumor (MPNST) is a rare soft tissue sarcoma, with an incidence in the general population of 1.46/100,000, originating from Schwann cells [7]. MPNST may occur sporadically in 45% of cases, while half of these tumors are associated with the NF1 [8]. Similarly to other soft-tissue sarcomas, surgical resection is currently the first-choice treatment. Nonetheless, it is often compromised by the large size of the tumors, usually due to late diagnosis, and by their proximity to complex nerve networks [1,9]. Due to its invasive growth and propensity to metastasize, MPNSTs have a poor prognosis and represent the primary cause of mortality for NF1-affected patients. The primary risk factors for developing MPNST rely on existing plexiform neurofibromas (PN), histologically benign tumors, which develop from Schwann cell biallelic *NF1* inactivation occurring in nearly 30–50% of NF1 patients [1,5,7,8]. A fundamental aspect of clinical practice for NF1 patients is monitoring PN for signs of transformation to MPNST, which occurs in 8–15% of patients [10]. However, malignant transformation from PN to MPNST is difficult to diagnose because of the impossibility of performing serial PN biopsies for NF1 patients who, in many cases, have multiple PNs and, furthermore, cross-sectional imaging cannot distinguish MPNST from PN with adequate specificity [11,12,13,14].

Despite the high incidence and mortality of MPNST in the NF1 population, screening for malignant transformation and monitoring of MPNST is challenging. Currently, there is no clear understanding of the molecular mechanisms involved in the tumor transformation of MPNSTs. Major efforts are focused on the use of next-generation sequencing techniques, which have identified additional mutations or copy-number alterations of genes such as *TP53*, *SUZ12*, *EGFR*, *CDKN2A*, and *TERT*, not present in the benign form of PN, representing alterations associated with advanced progression to the atypical neurofibroma (AN) and MPNST [6,15,16,17,18,19,20,21,22]. Therefore, high variability of the genomic context of MPNSTs, due to intra-tumor and substantial interpatient tumor heterogeneity, makes it difficult to identify a unique molecular signature [23,24].

In this work, we present a deep genomic characterization to implement an analytical model for the identification and evaluation of early molecular drivers in MPNST. Therefore, we performed whole exome sequencing (WES) on four tumor samples from a patient with MPNST-NF1-associated diagnosis—a benign sample, a primary tumor, a lung metastasis and a skin metastasis—and tested tumor-normal match approaches, with the aim of delineating the molecular alterations that contribute to the progression and metastasis of MPNST. Our results provide a basis for new studies, serving as a proof of concept, and reveal the necessity of systematically analyzing MPNSTs, combining pathology with genomic results, for improved early diagnosis.

## 2. Results

### 2.1. Patient Clinical Characteristics and Sample Collection

The primary objective of this pilot study was to determine the genomic signatures underneath malignant transformation and metastatic process from PN to MPNST using a genome-wide somatic approach. In our study, four DNA samples derived from formalin-fixed paraffin-embedded (FFPE) tissues were collected from a patient with MPNST-NF1-associated diagnosis, who presented conventional clinical features (>6 café-au-lait spots, Lisch nodules, axillary and inguinal freckling, cutaneous, subcutaneous and plexiform neurofibromas, optic glioma), and an atypical deletion in the *NF1* gene. The patient also developed lung metastasis and skin metastasis.

### 2.2. Somatic Whole-Exome Sequencing and Data Mining

**Figure 1:**
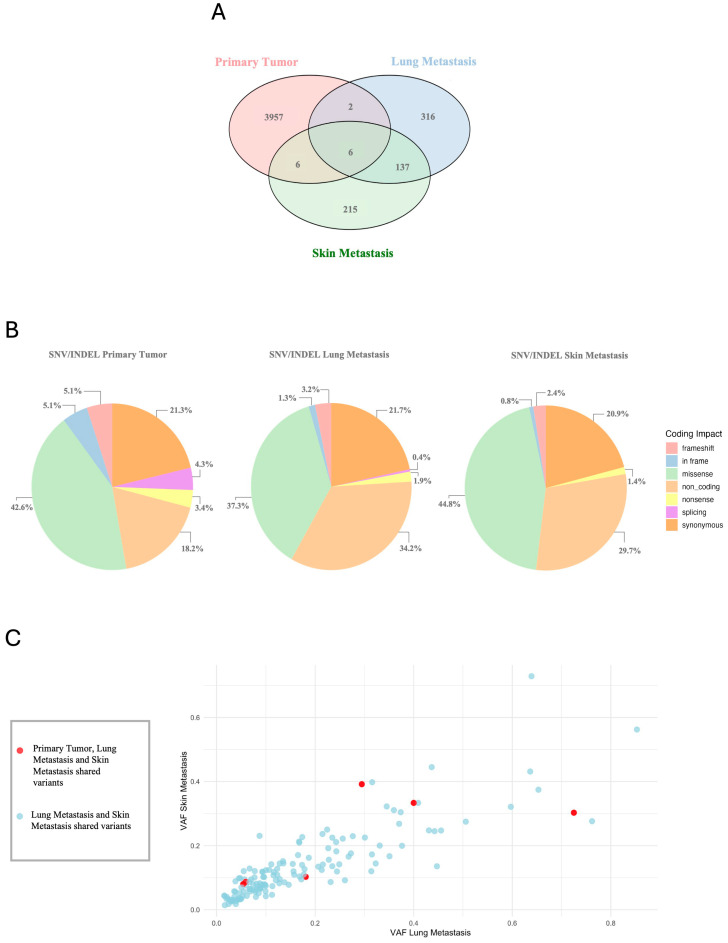
Genomic Variant Signature. (**A**) The Venn plot shows common and unique variants among primary tumor, lung metastasis, and skin metastasis, marking overlaps. (**B**) The pie charts illustrate the total number of variants identified in the different samples, according to the coding impact. (**C**) The VAF plot shows the distribution of variant allele frequencies (VAFs) in primary tumor, lung metastasis and skin metastasis (red), and lung metastasis and skin metastasis (blue).

To explore the molecular basis of MPNST, we performed WES on DNA samples extracted from benign tissue (selected by the pathologist from the plexiform neurofibroma sample), primary tumor sample diagnosed as MPNST, a sample from lung metastasis, and a sample from skin metastasis. The FASTQ files were loaded into the Varsome Clinical software v.12.3.2 to perform Tumor–Normal matching. After analyzing the WES data, we filtered out the variants according to the quality parameter (FILTER = PASS). We evaluated the impact of the genetic variants obtained at the coding sequence level, considering that the total number of changes differed significantly among the samples (Figure 1A): 3971, 461 and 364 variants for the primary tumor, the lung metastasis and the skin metastasis, respectively. As shown in Figure 1B, we observed differences in the frequency of variants among the distinct lesions. Missense (42.6%), synonymous (21.3%), and non-coding (18.2%) were the most common variants in the primary tumor. Frameshift variants (5.1%) were also found, along with in-frame (5.1%), nonsense (3.4%), and splicing variants (4.3%). For lung metastasis, the distribution of variants showed a reduction in missense variants (37.3%) and an increase in non-coding (34.2%) and synonymous (21.7%) variants. In addition, frameshift (3.2%), in-frame (1.3%), nonsense (1.9%), and splicing (0.4%) variants were not as prevalent in lung metastasis. Similarly to lung metastasis, for skin metastasis, the most frequent variants were missense (44.8%) and non-coding variants (29.7%), followed by synonymous (20.9%) and by nonsense variants (1.4%). Frameshift and in-frame variants were 2.4% and 0.8%, respectively, while splicing variants were not observed in this sample.

To identify any driver variants that could be responsible for tumor transformation, we executed a comparison between the genetic variants of the three samples, as shown in the Venn plot (Figure 1A). The genetic variants shared among all three samples were extremely limited, with a total of six variants across five different genes, namely *FAM86C1P*, *HLA-DRB5*, *KCNJ12*, *PSG5*, and *TMCO5A*. However, a higher number of significant variants emerged between the two metastatic samples. Instead, as expected, the comparison between genes involved in SNVs and Indels between the three samples (shown in Supplementary Figure S1A) exhibited a greater overlap than the comparison of variants. This may suggest that although the samples do not have the same mutations, they share an altered biological pathway.

A VAF plot (Figure 1C) displays the Variant Allele Frequency (VAF) for the common variants in lung and skin metastasis, along with the six variants that are shared in the primary tumor; a complete overview comprising the primary tumor is available in Supplementary Figure S1B. In particular, among the genes shared in the three samples, *KCNJ12* and *FAM86C1P* genes show significantly lower VAF in the primary tumor compared to metastases. Indeed, the variant in the *KCNJ12* gene has a VAF of 0.4 in lung metastasis and 0.7 in skin metastasis, while in the primary tumor the VAF is reduced to 0.2. Similarly, *FAM86C1P* shows a VAF of 0.3 in both lung and skin metastasis, but in the primary tumor it drops to 0.09. On the other hand, variants associated with the *PSG5*, *HLA-DRB5* and *TMCO5A* genes present an opposite trend, with a higher VAF in the primary tumor and lower in metastases. For the *PSG5* gene, we find two variants in which the primary tumor and the lung metastasis show a VAF of 0.3 and 0.05 for both variants, while in the skin metastasis the VAFs are 0.07 and 0.08, respectively. For *HLA-DRB5*, the VAF is 0.4 in the primary tumor, 0.2 in the lung metastasis, and 0.3 in the skin metastasis. Finally, in the *TMCO5A* gene, the VAF is 0.6 in the primary tumor, but is reduced to 0.3 in the lung metastasis and 0.4 in the skin metastasis, respectively.

### 2.3. Evaluation of the Pathogenicity and Clinical Significance of the Variants

**Figure 2:**
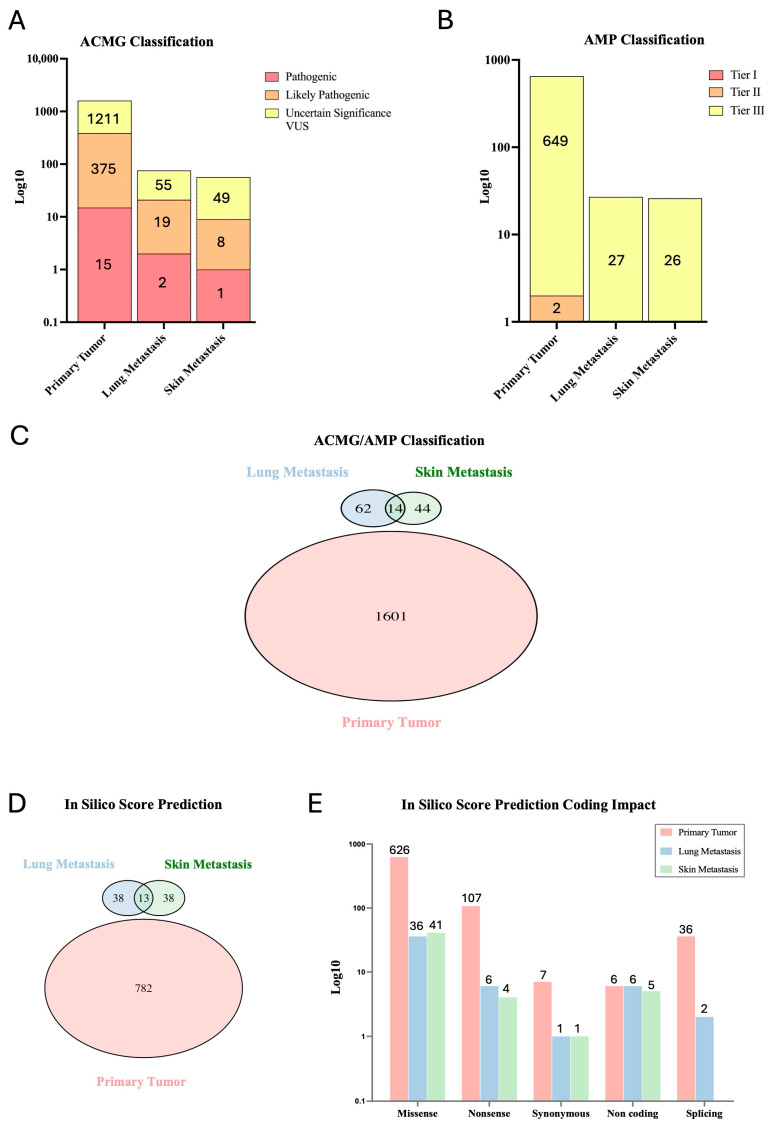
(**A**,**B**) The bar plots illustrate the number (log10-scaled) of candidate variants selected through the ACMG and AMP classification for the primary tumor, lung metastasis and skin metastasis. The selected variants are classified as pathogenic (red), likely pathogenic (orange) and uncertain significance (VUS in yellow) for ACMG, and Tier I (red), Tier II (orange) and Tier III (yellow) for AMP. The number within each bar represents the absolute number of the identified variants. (**C**) The Venn plot shows the distribution and intersection of unique and common variants between the primary tumor, lung metastasis, and skin metastasis, filtered for pathogenicity criteria ACMG/AMP. (**D**) In silico score prediction Venn plot of variants shared among the three samples and (**E**) bar plot of the total number (log10-scaled) of variants retained, with prediction score in the different samples according to the impact on the protein coding sequence. The number on the top of each bar represents the absolute count of variants.

In order to establish the biological relevance, we selected candidate variants (Supplementary Table S1) according to the pathogenicity ACMG (American College of Medical Genetics and Genomics) and AMP (Association for Molecular Pathology) criteria (Figure 2), to define those classified as pathogenic, likely pathogenic, and variants of uncertain significance (VUS) and those classified as Tier I (highly clinically significant), Tier II (clinically significant) and Tier III (uncertain clinical significance). Consistent with the ACMG guidelines, we found variants classified as pathogenic and likely pathogenic in all three samples. However, the greatest number of these variants was observed in primary tumor (15 pathogenic, 375 likely pathogenic and 1211 VUS), compared to lung metastasis (2 as pathogenic, 19 likely pathogenic and 55 VUS) and to skin metastasis (1 pathogenetic, 8 likely pathogenic and 49 VUS) (Figure 2A). By applying the AMP classification, primary tumor presents only 2 variants classified as Tier II and 649 variants classified as Tier III, while metastatic samples exhibit only Tier III-classified variants (27 in lung and 26 in skin metastasis samples) (Figure 2B). Thereafter, we compared the lists sorted by pathogenicity criteria (ACMG classes 3–5 and AMP tiers 1–3) to identify common variants among the three samples. The Venn diagram (Figure 2C) revealed that there were no common variants with the primary tumor, only within the two metastatic samples. Therefore, the six variants common to the primary tumor and metastasis (shown in Figure 1A) were lost, as they were classified as benign or likely benign.

Given the nature of the somatic variants, we repeated the analysis, generating a consensus list based on the prediction scores provided by the VarSome tertiary tool based on SIFT, CADD, BayesDel AddAF, BayesDel, REVEL and MetaSVM scores (Figure 2D). The consensus list was generated by including variants that displayed at least three out of six scores with a prediction of deleterious effect. Furthermore, for variants that did not have information on all six scores, we included those found to be pathogenic in the available scores. A total of 782 variants were found in the primary tumor, 51 variants in lung metastasis and 51 variants in skin metastasis (Figure 2D). This result is consistent with the previous one; analysis shows that variants classified as pathogenic by predictive scores are shared only by metastatic samples, and are not found in the primary tumor.

### 2.4. Comparative Analysis of the Genomic Profiles

**Figure 3:**
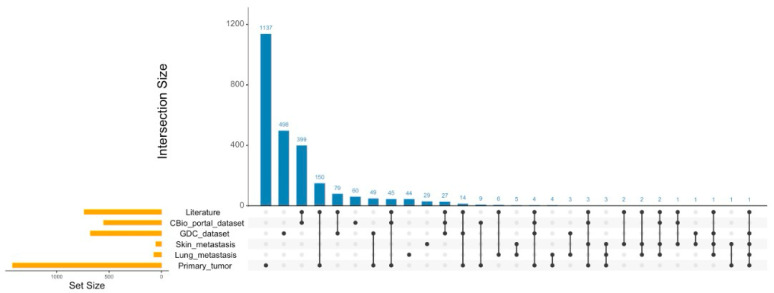
UpSetR graph. Visualization of intersections of the ACMG/AMP-based genes in primary tumor, lung metastasis, and skin metastasis samples with genes from the GDC-Data Portal, CBio-portal, and literature datasets [19,20,21,25,26,27,28,29]. It is important to note that UpsetR performs the intersection of all lists simultaneously. Therefore, the numbers obtained from pairwise comparisons may be lower, since genes common to more than two lists are not counted multiple times. This approach allows you to identify unique intersections between multiple datasets, providing a more complete view of the overlaps between different groups of genes, e.g., intersections between primary tumor and GDC are the sum of 49 (primary + GDC), 14 (primary + GDC + Lit), 4 (primary + GDC + Lit + CBio) and 1 (primary + metastasis + GDC), for a total of 68, as reported in the text. The list of literature genes is available in Supplementary Table S2.

We analyzed the genes associated with the candidate variants sorted with the ACMG/AMP pathogenicity criteria. We performed a comparison with datasets of genes from GDC Data Portal and cBio Portal, derived from patients affected with the nerve sheath tumors, and a list of genes associated with NF1-related MPNSTs from the scientific literature search (searched with the “NF1 or MPNST” MESH terms). By comparing the ACMG/AMP candidate gene list from the primary tumor with those datasets, we found 68, 61 and 217 genes in common with the GDC, cBioPortal datasets and the list of genes found in the scientific literature, respectively (Figure 3). In contrast, in the metastatic samples, the comparison with these datasets showed a minimal gene overlap. In the lung metastasis, the comparison with GDC revealed 5 genes in common, while that with cBioPortal and the scientific literature identified 2 and 12 genes respectively; in the skin metastasis, the comparison identified 3 genes in common with GDC, 6 genes with cBioPortal and 12 genes with the scientific literature.

### 2.5. Unveiling Key Pathways Underneath Genomic Signatures

**Figure 4:**
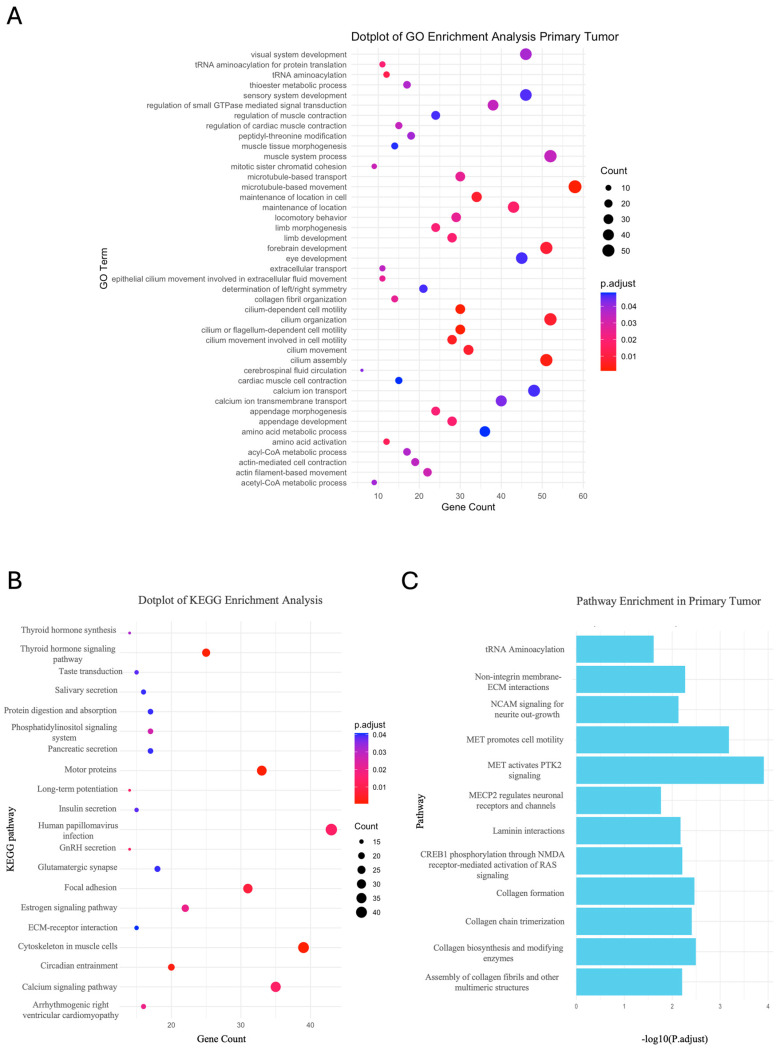
GO, KEGG and Reactome enrichment analysis of Primary Tumor sample. (**A**,**B**) Enriched GO-term dot plots. The size of the dots represents the number of genes in the significant gene list associated with the GO or KEGG terms. The color of the dots represents the adjusted *p*-values (Benjamini–Hochberg, BH). (**C**) Bar plots displaying the Reactome terms ordered by adjusted *p*-values (BH).

To define a biological significance of the candidate genes, we performed a pathway analysis using the information available in the Gene Ontology (GO), Kyoto Encyclopedia of Genes and Genomes (KEGG) pathway and Reactome databases, obtaining statistically significant results only for the primary tumor gene list. As shown in Figure 4, we found terms of GO associated with cell motility, cell division, cell signaling and metabolism (Figure 4A). KEGG analysis (Figure 4B) identified significant pathways associated predominantly with cell motility, transport and metabolism. Similarly, the analysis with Reactome (Figure 4C) also showed an enrichment of pathways associated with cell signaling and motility, metabolic processes and the structural organization of cells.

### 2.6. Pathogenic Gene Expression in Sarcoma and Metastatic Cancers: Key Indicators of Patient Survival

**Figure 5:**
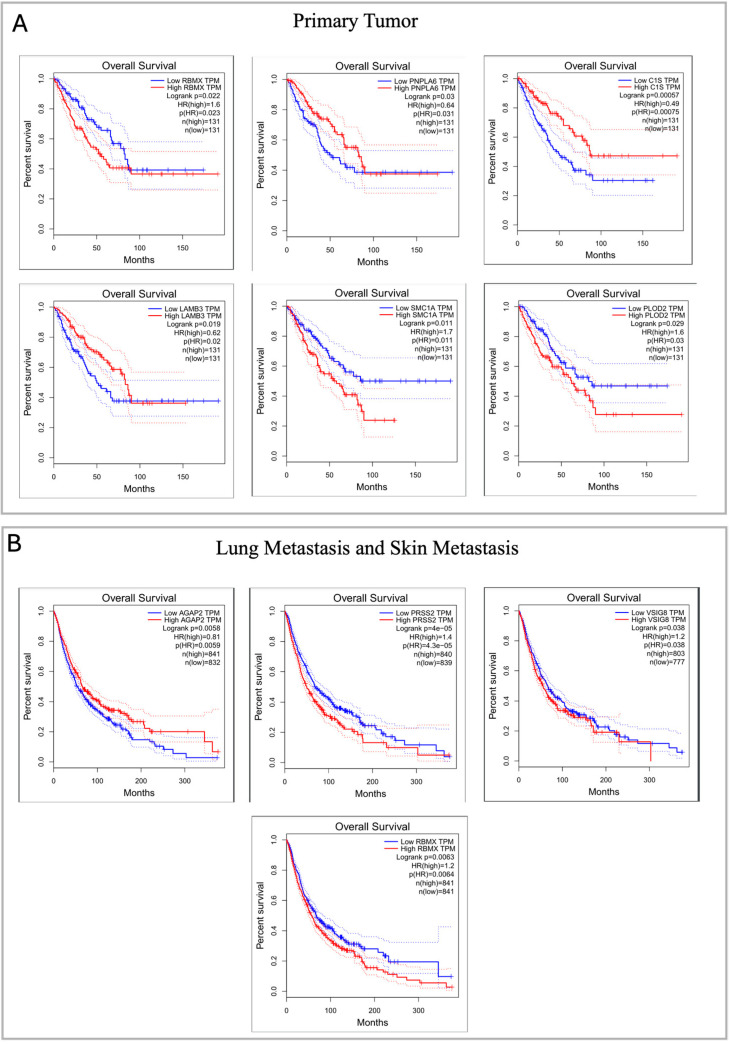
Kaplan–Meier Analysis. (**A**) Kaplan–Meier survival curves of patients affected with sarcoma (TGCA-SARC, n = 262) scored for different gene expression levels (LOW expression in blue and HIGH expression in red from the SARCOMA dataset) of the candidate genes identified in the primary tumor. (**B**) Kaplan–Meier survival curves obtained from transcriptomic data in sarcoma (TGCA-SARC, n = 262), lung cancer (TGCA-LUAD, n = 483) (TGCA-LUSC, n = 486) and skin cancer (TGCA-SKCM, n = 461) datasets, based on the expression of genes which are shared between the two metastatic samples.

Using The Cancer Genome Atlas (TCGA) database related to sarcoma samples (SARC), accessible via the GEPIA portal, we analyzed the correlation between the expression of genes classified as pathogenic in the primary tumor and the overall survival of patients with different expression of our candidate genes. Through Kaplan–Meier analysis, we observed that a change in expression of *RBMX*, *PNPLA6*, *C1S*, *LAMB3*, *SMC1A* and *PLOD2* was associated with lower and significant patient survival, as shown in Figure 5A. For the metastatic samples, we chose to perform Kaplan–Meier analysis on common genes filtered for pathogenicity criteria (ACMG/AMP) and integrating lung cancer (LUAD, LUSC) and skin cancer (SKCM) datasets from TCGA. Out of 14 common genes, only expression changes in *AGAP2*, *VSIG8*, *RBMX* and *PRSS2* were associated with reduced overall survival (Figure 5B).

### 2.7. Genomic Copy-Number Variant (CNV) Analysis

**Figure 6:**
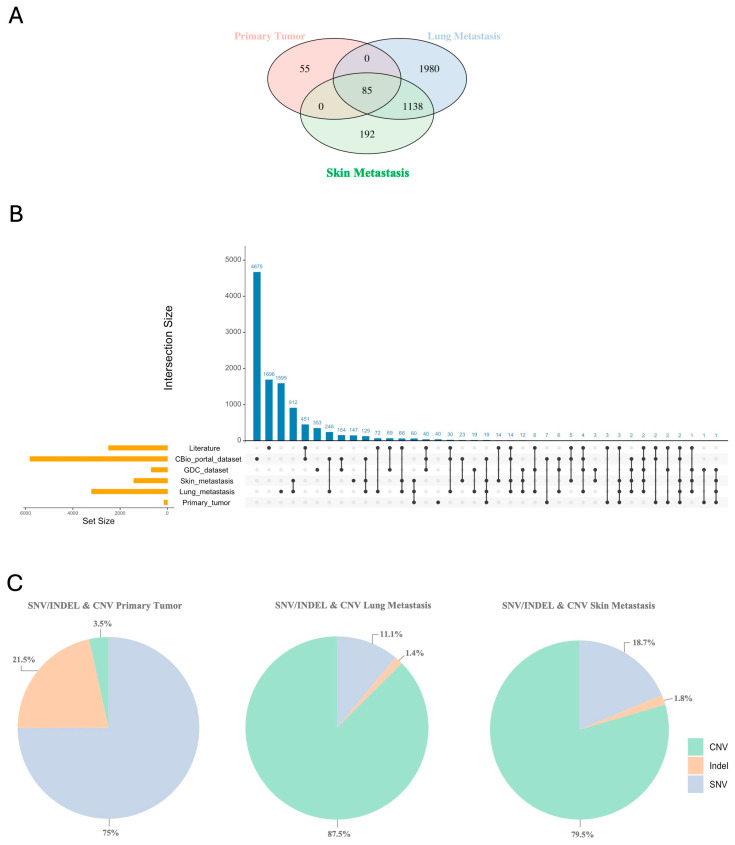
(**A**) Venn plot shows the distribution and intersection of unique and common genes identified in CNVs among the primary tumor, lung metastasis, and skin metastasis. (**B**) UpSetR graph display the intersections of CNV-associated genes in the primary tumor, lung metastasis, and skin metastasis samples with the genes from the GDC-Data Portal, CBio-portal, and literature datasets [19,20,21,25,26,27,28,29]. (**C**) Pie charts illustrate the percentage of variants identified in the different samples, according to the molecular type.

Finally, we used Varsome Clinical software (v12.3.2) to identify CNVs. The primary tumor exhibits a total of 67 CNVs, dissimilar metastases, which show 233 and 243 CNVs, respectively. In a similar way, we observed that the number of genes involved in CNVs in the primary tumor (n = 140) is significantly lower than that observed in lung and skin metastasis (n = 3203 and n = 1415, respectively). Although no point mutations were found in genes commonly associated with MPNST, CNVs were detected in the *SUZ12* and *TP53* genes in the primary tumor. Similarly to what was carried out for SNVs/Indels, we extracted the individual genes localized within the identified CNVs and performed a comparison between the different samples. A total of 85 genes were commonly deleted in each tumor sample as a driver signature (Figure 6A). We observed a significant number of deleted genes, identified as CNVs, which were shared across the primary tumor, the lung metastasis and the skin metastasis. However, the number of genes shared between the two metastatic samples is significantly higher (n = 1223) (Figure 6A).

By investigating the CNVs-related genes from the tumor samples, we confirmed that a high number of them are present in public datasets, such as the GDC and cBioPortal and the list of genes found in the scientific literature (Figure 6B).

Afterwards, we examined the total number of variants between SNVs, small Indels and CNVs for each sample, calculating their percentage (Figure 6C). In the primary tumor, we observed a high percentage of SNVs and Indels, accompanied by a low percentage of CNVs. In contrast, metastatic samples showed a low percentage of SNVs and Indels but a high percentage of CNVs. Therefore, although metastases lose some of the SNVs and Indels present in the primary tumor, they acquire a high number of CNVs, which reflects a different genetic evolution mechanism compared to the primary tumor.

**Figure 7:**
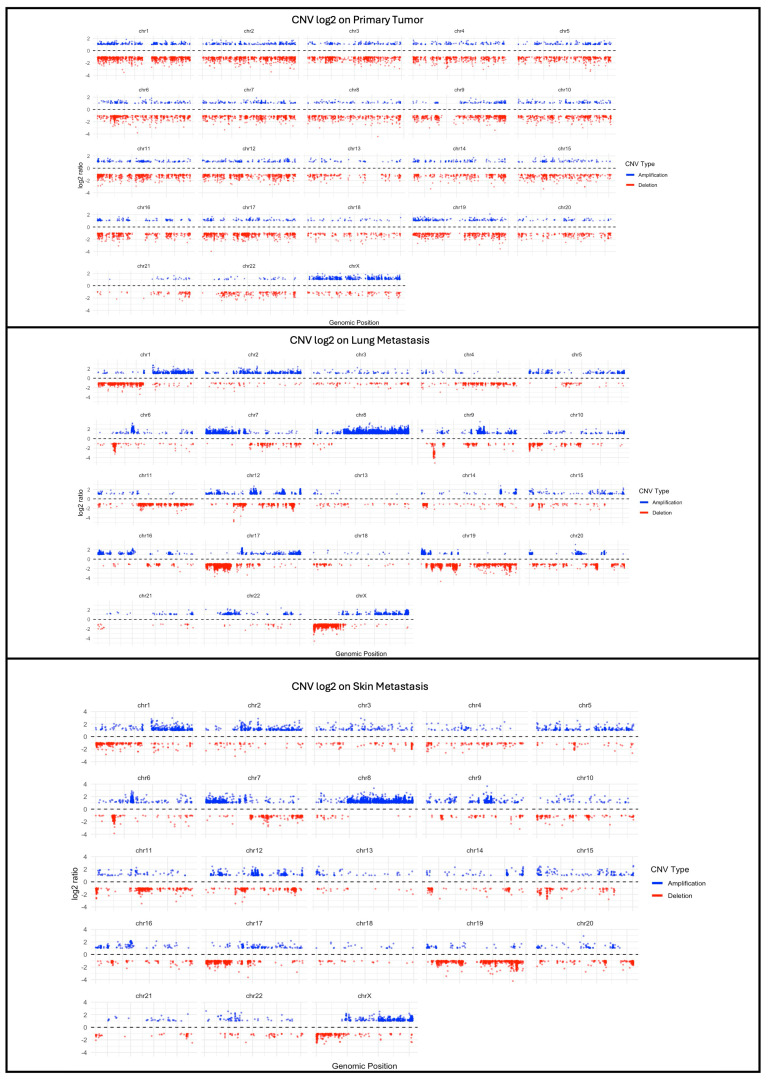
CNV profiling from primary tumor (**top**), lung metastasis (**middle**) and skin metastasis (**bottom**) performed with CNVkit. The variations in Log2 Ratio (on the y axis) indicate alterations in the number of genomic copies on a logarithmic scale, and are calculated as the log2-scaled ratio between the number of observed and expected copies in a sample. Log2 values greater than 1 (log2 > 1) represent amplifications of more than two copies; log2 values lower than −1 (log2 < −1) represent deletions of at least one copy.

Finally, we performed the calling of CNVs using the CNVkit, with the aim of clarifying the profiles of the number of CNVs in the three samples. As shown in Figure 7, in the primary tumor, amplifications and deletions are distributed relatively uniformly throughout the genome, with a small number of regions showing significant variations in their copy numbers (thus, having a log2 ratio > 2 or <2). Instead, the metastases show a more pronounced presence of amplifications and deletions, suggesting greater genomic instability. In particular, some regions of the genome show extreme alterations in the number of copies, with log2 ratio values of about 3 and −3, indicating an increase in the chromosomal instability as the tumor progresses towards the metastatic phase.

## 3. Discussion

In this study, we performed a genomic analysis of four samples from a single patient to detect driver genes associated with malignant peripheral nerve sheath tumor (MPNST) and to identify a genetic signature specific for sporadic MPNST. Moreover, we propose a data mining approach to unravel the genetic complexity of this tumor, allowing us to determine and validate pathogenic variants and target pathways associated with its onset and growth.

The investigation of whole exome sequencing data matching benign and tumor samples resulted in the identification of a different set of variants, as well as of biologically relevant genes and pathways associated with MPNST.

Both the primary tumor and the metastatic samples resulted in an expected core of missense variants, with an enrichment for non-coding variants in the lung and skin metastasis. A small amount of evidence is currently available regarding the contribution of non-coding variants to NF1-associated tumors, which was argued for the first time by Sedant and colleagues [30] and was just very recently reported by Liot and colleagues in Tenascin-X expression [31] and by Tritto and colleagues in the Antisense non-coding RNA in the INK4 locus (ANRIL) [32]. However, the contribution of non-coding variants to metastasis is increasingly observed in several types of cancers, such as adenocarcinoma [33], gastric cancer [34] and prostate cancer [35]. Hence, our results are aligned with this observed trend and may shed light on novel targets for a specific MPNST signature. The tumor samples showed significant differences in the number of genes harboring pathogenic or likely pathogenic single-nucleotide variants. On the one hand, the primary tumor sample displayed the highest number of genes having a great overlap with those already reported to be associated with MPNST, such as the MET Proto-Oncogene, Receptor Tyrosine Kinase (*MET*), the Hepatocyte Growth Factor (*HGF*), and others. On the other hand, the limited knowledge currently available on MPNST metastasis was evident in the small number of genes already reported to be associated with MPNST, in other studies. Among them, five genes were shared between the two metastases: the DAZ-associated protein 1 (*DAZAP1*), the Lysine Methyltransferase 2C (*KMT2C*), the Mucin 12 (*MUC12*), the Myosin heavy chain 9 (*MYH9*), and the Supervillin (*SVIL*); these genes were already identified as taking part in metastasis progression in different types of cancer [36,37]. Moreover, the large number of variants identified in the primary tumor is likely attributed to its high heterogeneity, which may reflect the generation of numerous tumor clones. These clones, in turn, are likely selected in metastasis for their increased aggressiveness.

Given the current limited knowledge of molecular mechanisms and genes involved in MPNST pathogenesis, the comparison of candidate genes selected for pathogenicity criteria (ACMG and AMP) in the primary tumor with those found in the literature has allowed the identification of not only genes with recurrent mutations, such as *NF1*, but also other genes potentially involved in the pathogenesis of the MPNST, including ATRX chromatin remodeler (*ATRX*) [20,27], Picolo Presynaptic Cytomatrix Protein (*PCLO*) [21,27], Low-Density Lipoprotein Receptor-Related Protein 1B (*LRP1B*) [20,21,25,27], Breast Cancer 2 (*BRCA2)* [21], Erb-B2 Receptor Tyrosine Kinase 4 (*ERBB4*) [28], and Laminin Subunit Alpha 2 (*LAMA2*) (Laminin Subunit Alpha 2) [20,21].

Except for *ERBB4*, known to promote the pathogenesis of malignant peripheral nerve sheath tumor (MPNST) through mechanisms independent of Ras, [38], all the remaining genes are well-known for taking part in types of cancer [39,40,41,42,43], and could be promising targets for future studies.

Moreover, our investigation was able to identify not only short variants, but also copy-number variations. CNV analyses reflected the tumor environment and its genomic instability: these variations were mostly identified in lung and skin metastasis, rather than in the primary tumor. In the primary tumor, CNVs were identified only in two genes that were already reported to be associated with MPNST: the Tumor protein 53 (*TP53*) and the SUZ12 polycomb repressive complex 2 subunit (*SUZ12*) [20]. Despite the well-known role of TP53 in different types of cancer, the role of *SUZ12* was assessed in the last decade: alterations and losses in the polycomb repressive complex 2, of which *SUZ12* is a key component, cause signaling dysregulation and contribute to oncogenesis through cell proliferation and growth [44,45]. A great number of CNVs were observed in metastatic tumors, characterized by higher levels of instability and underlining tumor aggressiveness. A comparison between primary and metastatic tumor samples resulted in the identification of an opposite trend: the primary tumor shows an increased number of SNVs and small INDELs, rather than of CNVs; the metastatic tumors show an increase in the number of CNVs. According to various studies [46,47,48], a high number of CNVs and a lower number of short variants (either SNVs or and small INDELs) in the metastases suggest a higher genomic plasticity during disease progression, which might reflect different mechanisms of tumor evolution.

Furthermore, by investigating the type of molecular changes, we observed a great number of synonymous variants and variants with a low level of impact on the protein sequence, particularly in the primary tumors. Thereby, we tested different criteria based on ACMG pathogenic guidelines, AMP druggable variants, and predictive scores, to select candidate variants which may represent the tumor driver signature to validate in the next step of functional analysis.

Novel candidate genes were also tested by survival analysis, mediated by expressional changes in genes carrying only pathogenic or likely pathogenic variants. The expressional changes of nine genes were found to significantly decrease patient survival in all the tumor samples, highlighting the reliability of our candidate gene list as putative drivers of pathogenic mechanisms. In the primary tumor, these changes were triggered by six genes, namely Patatin-Like Phospholipase Domain Containing 6 (*PNPLA6*), Complement C1s (*C1S*), Laminin Subunit Beta 3 (*LAMB3*), the Structural Maintenance Of Chromosomes 1A (*SMC1A*), and the Procollagen-Lysine,2-Oxoglutarate 5-Dioxygenase 2 (*PLOD2*); in the metastatic samples, these genes comprised the Arf GAP With GTPase Domain, Ankyrin Repeat And PH Domain 2 (*AGAP2*), the V-Set And Immunoglobulin Domain Containing 8 (*VISG8*), and the Serine Protease 2 (*PRSS2*) genes; the RNA Binding Motif Protein X-Linked (*RBMX*) was common to the primary and metastatic tumors. Apparently, these genes seemed unrelated to either NF1 or MPNST; however, both their biological roles and functional associations made them novel candidates for MPNST. Some of them are involved in the development of the nervous system and in neuron growth and differentiation, such as *PNPLA6* and *AGAP2*; others take part in the defense of our organisms, such as *C1S*, *AGAP2* and *PRSS2* in the immune system, and *SMC1A* and *RBMX* in DNA damage and repair. From a pathological perspective, all these genes were already reported to be associated with disorders similar or related to NF1 and MPNST. Among these, *PNPLA6* and *RBMX* are described in polyneuropathies and neurological disorders. Among the genes resulting from the primary tumor, *LAMB3* was already associated with nervus and brachial plexus neoplasms, conditions closely related to MPNST. On the other hand, genes resulting from skin and lung metastases were already reported to be associated with diseases in the respective tissues, such as *AGAP2* with skin cancer [49] and *VSIG8* with respiratory syndromes [50]. However, other candidate genes that do not significantly impact on patients’ survival were observed to be present in genomic sarcoma databases, representing a genomic scar of the MPNST. This significant overlap between the genomic profile of the primary tumor and the various datasets highlights the congruence between our sample and what is currently available on peripheral nerve sheath tumor, supporting the biological relevance of our observations. On the other hand, the low number of genes present in the dataset in common with the metastatic samples may be due to data being obtained specifically from nerve sheath tumor samples, which are therefore more relevant for the primary tumor than for metastasis.

Pathways analysis confirmed the biological relevance of all the identified genes, either already reported to be associated with MPNST or novel findings. Among the most significant results, a cluster pointed at physiological cellular functions, such as cell motility and cell division. This is complemented by the results of the gene set enrichment analysis (GSEA) conducted by Stricker et al., which demonstrated an enrichment of gene sets related to the G2-M checkpoint and chromosome condensation on MPNSTs [51], and by Pemov et al.2017 [52], which underlined critical pathways involved in processes such as the start of DNA replication, filament elongation, telomere maintenance and cell proliferation and differentiation already in the plexiform neurofibroma. These functions are also fundamental for tumor growth and development: when the functions of the associated genes are altered, these pathways become oncogenic, and thus might represent an oncogenic signature specific to the MPNST primary tumor or metastasis. The pathway of actin cytoskeleton regulation and focal adhesions plays a crucial role in tumor migration and metastasizing. However, it seems that these biological and cellular processes are already altered in the plexiform neurofibroma, as demonstrated by Grit et al., due to methylation events that alter the expression of the genes involved [53]. Moreover, cell signaling and metabolism-related functions are associated with the candidate genes, confirming the importance of the metabolic switch observed in more aggressive tumors.

## 4. Materials and Methods

### 4.1. Sample Collection and DNA Extraction

Four samples were collected from the same patient, including three tumor samples (a primary tumor, a lung metastasis and a skin metastasis) and one benign sample (selected from the benign area of plexiform neurofibroma). The gDNA was purified starting from formalin-fixed paraffin-embedded (FFPE) samples obtained at surgical resection, using the Magcore DNA FFPE One-Step Kit (Code 405). Fragmented gDNAs were tested for size distribution and concentration using an Agilent Tapestation 4200 and Qubit dsDNA High Sensitivity kit.

### 4.2. Whole Exome Sequencing (WES)

The NGS library was executed using the Agilent Sure Select XT HS2 all exon v8 kit (Agilent, Santa Clara, CA, USA), following the manufacturer’s instructions. For samples with a low DNA Integrity Number (DIN), the first fragmentation step has been optimized by reducing the enzyme fragmentation time. The samples were then sequenced on an Illumina NextSeq550Dx, which generated paired-end reads of 151 bp.

### 4.3. Sequencing Data Analysis

Candidate somatic variants, consisting of point mutations, insertions, and deletions were sorted out with Varsome Clinical Software v.12.3.2 (Saphetor, Lausanne, Switzerland). Briefly, an alignment filter was pursued to exclude quality failed reads, unpaired reads, and poorly mapped reads in the tumor. A base quality check was used to limit inclusion of bases with reported Phred quality score > 30 for the tumor and >20 for the normal. The variant calling was performed by comparing each tumor sample with the benign specimen (match tumor–normal) using default parameters (Reference genome: GRCh38/hg38). The final output is thus characterized by a VCF file in which variants in common for the benign and tumor samples have been filtered out (such as germinal variants or sequencing errors) and only somatic variants of the tumor have been selected. Candidate somatic changes were further filtered, based on gene annotation, to determine those lying in protein-coding regions. Functional consequences were predicted using the Varsome Clinical software v.12.3.2 (Saphetor, Lausanne, Switzerland) tertiary analysis tool, examining data from public databases such as 1000 Genomes, dbSNP, COSMIC, and ClinVar. Relevant somatic variants have been prioritized according to the pathogenicity score or the druggable classification in Tier, based on ACMG/AMP criteria (Supplementary Table S1) [54]. Finally, intronic and silent variants were excluded, while mutations resulting in missense mutations, nonsense mutations, frameshifts, or splice-site alterations were kept. A manual visual inspection phase was utilized to further remove artifactual alterations. The VAF plot was generated using Plotly v4.9.3 in the R environment (available from https://plotly-r.com). CNVs were identified and analyzed using Varsome Clinical software v.12.3.2 with default parameters. Finally, to determine the profiles of the alterations in the number of copies, the CNV calling was also performed using the CNVKit version 0.9.11 [55]. The output was then visualized using ggplot2 v3.5.1 [56] (https://ggplot2.tidyverse.org).

### 4.4. Driver-Gene Identification and Kaplan—Meier Analysis

We downloaded data from the TCGA-SARC from Genomic Data Commons (GDC Data Portal) (https://portal.gdc.cancer.gov/) and the dataset relative to nerve sheath tumor from the cBio Cancer Genomics Portal (https://www.cbioportal.org/) to identify potential driver genes. Both GDC Data Portal and cBio Portal are open-access resources for interactive exploration of multidimensional cancer genomic datasets, currently providing access to data from different cancer samples [57,58].

The list of genes in the literature was created by conducting a bibliographical search on PubMed, using the MESH terms “MPNST” and “NF1”. Papers mentioning the most frequently mutated genes were selected; in addition, extensive research was carried out on the Supplementary Materials to assess further genes whose variants could have a significant impact on MPNST and that were not mentioned in the main text of the articles. When this information was not available, we downloaded the available raw data from public repositories) and applied the same analytical pipeline that was used with our samples [19,20,21,25,26,27,28,29]. Kaplan–Meier survival curves were generated using GEPIA (http://gepia.cancer-pku.cn/) on the TCGA-SARC, TCGA-LUAD and TCGA-LUSC RNA-seq expression dataset, with two-tailed 95% confidence intervals [59].

### 4.5. Enrichment and Pathway Analysis

Gene Ontology (GO) and pathway enrichment analysis was performed using Cluster Profiler v.4.0 [60], selecting statistically significant terms and pathways (*p*-value < 0.05). The plots of pathways and enrichment were visualized by R ggplot2 package v.3.5.1 [56] (https://ggplot2.tidyverse.org). Visualization of intersecting sets was performed with UpSetR Version 1.4.0 (R package) [61].

## 5. Conclusions

In summary, this work presented a deep genomic characterization of MPNST samples that underwent whole exome sequencing. This study represents an omni-comprehensive genomic approach to identify early molecular drivers in MPNST. Despite the small number of analyzed samples and the limits of using tissues from a single patient, this approach offers a proof of concept which might represent a standard procedure to investigate NF1-related tumors. To date, little knowledge exists regarding MPNST in terms of both molecular mechanisms and genetic variability. To unravel this rare complex tumor, different worldwide research centers gathered in the Genomics of MPNST (GeM) Consortium, to understand the genetic of MPNST from a multi-omics perspective [24]. Overall, this investigation confirmed previously reported genes, as well as identifying novel candidate genes, proposing a prognostic oncogenic signature specifically tailored for MPNST.

## Data Availability

The original data presented in the study are available on Synapse with the following project identifier: syn63432900.

## Supplementary Materials

The following supporting information can be downloaded at: https://www.mdpi.com/article/10.3390/ijms251910822/s1.
