# Supplementary material for: Proof of Concept for Genome Profiling of the Neurofibroma/Sarcoma Sequence in Neurofibromatosis Type 1
Source: Int J Mol Sci. 2024 Oct 9;25(19):10822. doi: 10.3390/ijms251910822 (PMC11476461; doi:10.3390/ijms251910822)

Supplementary Figure S1: (**A**) Venn plot shows the distribution and intersection of unique and shared genes involving in SNVs and Indels (FILTER = PASS) between the primary tumor, lung metastasis, and skin metastasis samples. (**B**) The 3D VAF plot shows the distribution of Variant Allele Frequencies (VAF) in primary tumor, lung metastasis and skin metastasis (FILTER = PASS).

A

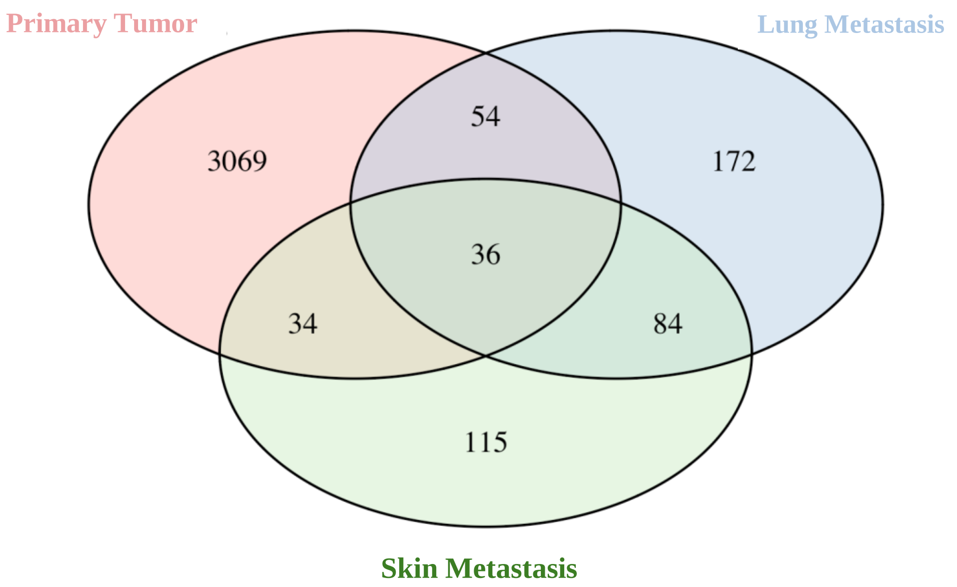

B

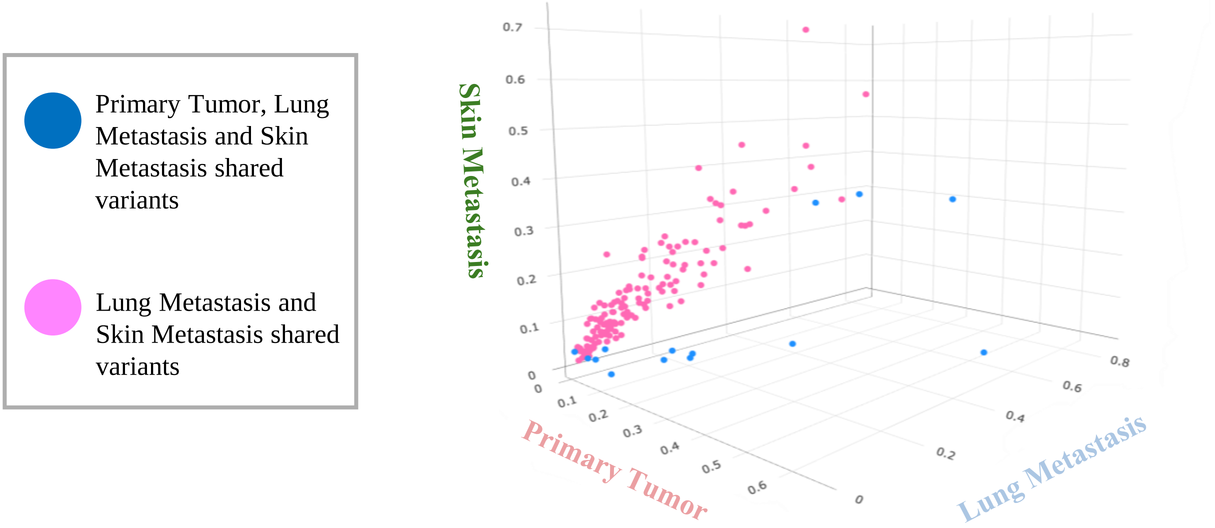

Supplement: Supplementary file 1 [file ijms-25-10822-s001.zip › Supplementary Figure S1.pdf]
